# Turbulence mediates marine aggregate formation and destruction in the upper ocean

**DOI:** 10.1038/s41598-019-52470-5

**Published:** 2019-11-07

**Authors:** Marika Takeuchi, Mark J. Doubell, George A. Jackson, Misuzu Yukawa, Yosuke Sagara, Hidekatsu Yamazaki

**Affiliations:** 1 Department of Ocean Sciences, Tokyo University of Marine Science and Technology, Minato-ku, Tokyo, Japan; 2 College of Science and Engineering, Flinders University, Adelaide, SA 5001 Australia; 3 South Australia Research and Development Institute, Aquatic Sciences, West Beach, SA 5024 Australia; 4 School of Biological Sciences, University of Adelaide, Adelaide, SA 5005 Australia; 5 Department of Oceanography, Texas A&M University, College Station, TX USA

**Keywords:** Ecology, Ocean sciences

## Abstract

Marine aggregates formed through particle coagulation, large ones (>0.05 cm) also called marine snow, make a significant contribution to the global carbon flux by sinking from the euphotic zone, impacting the Earth’s climate. Since aggregate sinking velocity and carbon content are size-dependent, understanding the physical mechanisms controlling aggregate size distribution is fundamental to determining the biological carbon pump efficiency. Theoretical, laboratory and *in-situ* studies of flocculation have suggested that turbulence in the benthic boundary layer is important for aggregate formation and destruction, but the small number of field observations has limited our understanding of the role of turbulence on aggregation processes in the ocean surface layer away from energetic boundaries. Using simultaneous field observations of turbulence and aggregates, we show how aggregate formation, destruction, morphology and size distribution in the ocean surface layer (10–100 m) are mediated by interactions between turbulence and aggregate concentration. Our findings suggest that turbulence enhances aggregate formation up to a critical turbulent kinetic energy dissipation rate of 10^−6^ (W kg^−1^), above which the smallest turbulent eddies limit aggregate size.

## Introduction

Marine aggregates range in size from approximately 1 µm to several centimetres^1^. Formed in the sunlit upper layers of the ocean and composed predominantly of organic material^1–3^, aggregates sink as a constant drizzle to the deep ocean^3^, exporting energy and acting as hotspots of microbial activity and biogeochemical transformations along the way^4^. Aggregate formation occurs through the collision and adhesion of smaller particles into larger particles and is driven by three main physical processes^5,6^. Brownian motion controls the collision of small particles (<1 µm). Differential sinking involves faster settling particles overtaking and colliding with slower settling particles and dominates for particle sizes between about 1 and 100 µm. Turbulent shear dominates the interactions between larger particles^5^ (>100 µm). Since large aggregates have increased sinking velocity and carbon content relative to small aggregates^7^, the mechanisms controlling aggregate size distribution in the upper ocean have important consequences for determining the transport of carbon to the deep ocean^8,9^.

The role of turbulence on aggregates has been investigated theoretically and experimentally^10^ over a range of flow conditions and materials, usually using idealized shear models and uniform spheres as source particles^11,12^. However, our understanding of the influence of turbulence on aggregates in the upper ocean interior has remained constrained due to a lack of direct observations. Early laboratory^13^ and modelling^14^ studies indicated that turbulence was important for both aggregation and disaggregation, consistent with the conceptual view of sedimentation processes in estuarine systems^15^. Subsequent observations made in the sediment-rich benthic boundary layer revealed that our understanding of particle disaggregation remains uncertain^16^ with minimal influence of turbulence detected on aggregate size, even though modelling of disaggregation processes in the bottom boundary layer predicted aggregate breakup under strong turbulence^14^.

In comparison to early experimental and modelling studies^10–17^, a laboratory study^18^ using natural aggregates collected from the ocean’s surface layer (10–15 m depth) found that turbulent kinetic energy dissipation rates as strong as 10^−4^ W kg^−1^ did not cause aggregate breakup. Estimates of drag forces on falling aggregates^19^ have further suggested that sinking-induced stresses may be more effective in causing aggregate breakup than turbulence. The conclusion from these studies^18,19^ was that disaggregation by turbulence was relatively unimportant in the upper ocean. Experimental^11^ and numerical studies^12^ investigating the collision of small particles (<~100 µm) have since shown that turbulence initially enhances aggregation, but disaggregation becomes increasingly important in controlling aggregate size distribution as the system ages and aggregates grow. More recent observations of aggregates made in the coastal benthic boundary layer^20,21^ and energetic tidal channels^22,23^ have shown that turbulence may indeed cause aggregate breakup, thereby limiting the size distribution of aggregates formed, reinstating the likely importance of disaggregation by turbulence.

The general applicability of these studies to understanding aggregation processes in the upper layers of the ocean remains uncertain for several reasons. First, the biological composition of marine aggregates that affects aggregation and disaggregation processes is known to be highly sensitive to changes in the ambient environmental conditions and the methods used for collection^24^. For example, the stickiness of diatoms following collection from the field has been shown to increase due to nutrient and light limitation^25–27^. Aggregates become compacted under even most gentle collection methods, potentially leading to stronger bonds^24^, which may be the reason aggregates survived the strong turbulence generated in the laboratory experiments^18^. Aggregates in the benthic boundary layer tend to be much smaller than the marine aggregates observed in the upper ocean water column, in part because their composition contains a higher density of the minerals and sediments^28^. In comparison, the composition of aggregates formed in the ocean surface layer contains an increased fraction of organic material, including living and dead phytoplankton^1,2^, fecal pellets^3^ and extracellular polymeric substances (EPS)^29^.

Another important difference between laboratory experiments, energetic coastal environments (e.g., bottom boundary layer, tidal channels) and the upper water column of the open ocean is the intensity of turbulence. Turbulent kinetic energy dissipation rates in the upper water column rarely exceed 10^−6^ W kg^−1^, except in the top few meters of surface layer when breaking surface waves^30,31^ generate strong turbulence. Similarly, kinetic energy dissipation rates can far exceed 10^−5^ W kg^−1^ in laboratory experiments and in the bottom boundary layer of coastal environments, where waves and currents can generate intense near-bed turbulence^30^. As a result, the relationship observed between turbulence and aggregates in highly localized bottom boundary layers^20,21^ and energetic coastal waters^22,23^ are not likely to be representative of processes occurring in the water column interior of the upper ocean that occupies most of the world ocean.

These uncertainties support the necessity of field measurements in the upper ocean to develop our understanding of the relationship between turbulence and aggregates and its implications for the biological pump under climate change^32,33^. In the present study, we collected simultaneous measurements of turbulence and aggregates in the upper ocean (~10–100 m) away from energetic coastal environments. We explore how aggregate size and other related properties, such as morphology and volume concentration, are affected by turbulence in the sunlit upper layer of the world ocean where particles are formed by primary production.

## Methods

We made non-disruptive measurements of turbulence and aggregates in the upper ocean water column, between the surface and 100 m. Measurements were made during 10 campaigns and multiple seasons in coastal and offshore waters of Japan.

Microscale variations in temperature and turbulent velocity were measured with a free-fall microstructure profiler (TurboMAP-L, JFE Advantech Co., Ltd.)^34^ at a sample rate of 512 Hz and fall-speed of ~0.5 m s^−1^. We estimated the turbulent kinetic energy dissipation rate (*ε*, W kg^−1^) by integrating the turbulent velocity shear spectrum obtained from the shear probe over 2 second segments (~ 1 m) from approximately 1 cycle per meter to half the Kolmogorov wavenumber ((*ν*^3^/ε)^−^^1/4^)^35,36^, where *ν* is the kinematic viscosity of seawater. A correction was made to recover the unresolved variance^37^ using the Nasmyth empirical spectrum^38^. The size of the shear probe that measures turbulent velocity was designed to resolve an expected minimum level of ε ~ 10^−10^ W Kg^−1^^39^ under the assumption of isotropic turbulence^40^. Although turbulence may not be isotropic when ε is low, axisymmetric turbulence theory that accounts for stratification effects on turbulence indicates the error associated with use of the isotropic turbulence theory is less than 35%^41^. Therefore, ε estimates based on isotropic turbulence theory are a reasonable approximation to its true value. To avoid contamination by vessel-generated turbulence, we discarded ε observations obtained within 10 m of the surface. Increases in the 1 m scale turbidity or *ε* were used to detect the presence of bottom boundary layer in waters less than 100 m deep. Since these signals were typically detected much closer than 10 m from the bottom, we discarded observations made within the bottom 10 m of all profiles to avoid contaminating water column observations with those from benthic boundary layers.

A mini CMOS camera (DSL II 190, Little Leonard Inc.)^34^ mounted on TurboMAP-L collected images of aggregates at a sampling rate of 5 Hz simultaneously with shear observations. Processed images had a field of view of 2 cm × 2 cm and a pixel resolution of 59 µm^34^. Streaked images were identified by assessing the 2D image spectrum using a 2D Fast Fourier Transform (2D FFT)^34,42^. The 2D spectrum is a symmetric circle when images are not smeared, whereas asymmetry is seen in the 2D spectrum of streaked images. To test for asymmetry, two perpendicular sets of 1D spectra were chosen and the ratio of variance for each wavenumber was calculated for each perpendicular pair. The variance ratio is approximately 1 in unstreaked images, with images rejected from further analysis if the average variance ratio for one perpendicular pair exceeded 1.5 or 1/1.5. This criterion assesses smearing across all aggregates imaged in the field of view and minimizes the rejection of images which contain rare individual long and thin aggregates.

Individual aggregates were then approximated as ellipses using the *regionprops* function in MATLAB (Mathworks Inc.) to determine major (*MajAL*) and minor (*MinAL*) axis lengths and equivalent spherical diameters (*ESD*). To focus on the larger size fraction of aggregates expected to be influenced by turbulence^5^, only objects with *MajAL* > 0.03 cm were considered to be aggregates. Coincident high-resolution fluorescence microstructure profiling that resolved millimetre scale changes in chlorophyll-*a* fluorescence showed extremely strong signals where aggregates were seen^34^, implying that aggregates captured by the DSL camera contained live phytoplankton. Additional laboratory tank experiments using particles of known size were also conducted to confirm that unfocused particles and streaked images were removed by the size threshold and 2D spectrum criteria. In total, 57,669 images collected over 148 profiles were retained. A total of 1,269,978 aggregates were identified; among them 1,103,412 aggregates were observed for *ε* < 10^−6^ W kg^−1^ and 166,566 aggregates for *ε* > 10^−6^ W kg^−1^.

Relationships between turbulence and aggregates were then examined using 10 m scale average properties that included, the average turbulent kinetic energy dissipation rate ($\bar{\varepsilon }$, W kg^−1^), total aggregate volume concentration (*V*_*agg*_, ppm), aggregate minor axis length ($\bar{{M}{i}{n}{A}{L}}$, cm), major axis length ($\bar{{M}{a}{j}{A}{L}}$, cm), equivalent spherical diameter ($\bar{{E}{S}{D}}$, cm) and aspect ratio ($\bar{{A}{R}}\,=\,\frac{\bar{{M}{a}{j}{A}{L}}}{\bar{{M}{i}{n}{A}L}}$), where the over bar represents the 10 m scale mean value. Since the imaging system provides a 2D image of a 3D object, differences in aggregate size due to orientation are expected to be reduced by the use of 10 m scale average metrics. The volume of an individual aggregate is calculated as $\frac{\pi }{{6}}{{E}{S}{D}}^{3}$, where *V*_*agg*_ is the fraction of volume occupied by aggregates and is expressed in cm^3^ m^−3^, equivalent to parts per million (ppm).

Aggregate number spectra^43^ (*n*) were used to describe the size distribution of aggregates. The number of aggregates (*ΔN*) in logarithmically increasing *MajAL* bins of average size *d* was divided by the bin width (*∆d)* and the sample volume to construct a number spectrum. Any *ΔN* < 10 was discarded before computing *n*. For each spectrum, a bilinear relationship was fit to log(*n*) as a function of log(*d*) to obtain values for the inflection point (*L*_*int*_) and the slope below (slope 1, small aggregates) and above (slope 2, large aggregates) the *L*_*int*_. The mean sum of squared error of each fit was then calculated. Different *L*_*int*_’s were then selected at intervals of ∆d either side of the first *L*_*int*_ and the slopes determined. The final accepted *L*_*int*_ and slopes were those with the smallest mean sum of squared error.

Finally, the distribution of aggregate volume as a function of the ESD size expressed as the normalised volume distribution *nVd*^43,44^ was estimated for each order of magnitude of $\bar{\varepsilon }$ of between 10^−10^ < *o(*$\bar{\varepsilon }$*) < *10^−5^ W kg^−1^. For each *o(*$\bar{\varepsilon }$*)* interval, the number of aggregates in logarithmically increasing *ESD* bin sizes (*d*) was divided by the bin width (*∆d)* and sample volume to construct an *ESD* number spectrum. *ESD* number spectra were multiplied by *V* = $\frac{\pi }{{6}}{{E}{S}{D}}^{3}$ and *d* to obtain *nVd*, whereby the integral of *nVd* is equivalent to ${V}_{agg}=\int nV\,{\rm{d}}d\,=\int nVd\,{\rm{d}}(ln\,d)\cdot \,$

## Results and Discussion

**Figure 1:**
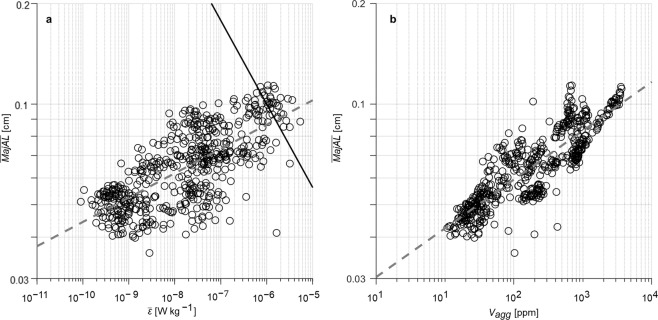
Changes in aggregate size with **(a)** turbulence intensity and **(b)** aggregate volume concentration. Changes in average major axis length ($\bar{{M}{a}{j}{A}{L}}$, cm) of aggregates (*n* = 567) with corresponding measures of: (**a**) average turbulent kinetic energy dissipation rate ($\bar{\varepsilon }$, W kg^−1^) and (**b**) total aggregate volume concentration (*V*_*agg*_, ppm). All values were calculated over 10-m depth intervals. The black solid line in (**a**) shows the Kolmogorov length scale and grey dashed lines in (**a**,**b**) indicate regression lines.

Values of $\bar{\varepsilon }$ ranged from 10^−10^ to 10^−5^ W kg^−1^ (Fig. 1a) and spanned the full range of naturally occurring turbulence intensities found in the upper ocean interior, away from energetic surface and bottom boundary layer regions^30,31^. Aggregate $\bar{{M}{a}{j}{A}{L}}$ ranged between 0.031 and 0.133 cm and log_10_ ($\bar{{M}{a}{j}{A}{L}}$) and was positively correlated with log_10_($\bar{\varepsilon }$) (Fig. 1a, *r*^*2*^ = 0.52, *n* = 567, *p < * < 0.001). The majority of ($\bar{{M}{a}{j}{A}{L}}$) were smaller than the size of the smallest turbulent eddies, here defined by the Kolmogorov length scale (*L*_*k*_ = (*ν*^3^/*ε*)^1/4^). Positive correlation between log_10_($\bar{{M}{a}{j}{A}{L}}$) and log_10_(*V*_*agg*_) (Fig. 1b, *r*^*2*^ = 0.74, *n* = 567, *p* ≪ 0.001) indicates that *V*_*agg*_ is also a crucial factor determining the size distribution of aggregates, since aggregate total volume is a measure of the number of particles available for coagulation^6^. Higher particle concentrations should increase coagulation rates, leading to larger particles, while sinking and disaggregation prevent particles from becoming indefinitely large. Multiple linear regression analysis showed that log_10_($\bar{\varepsilon }$) and log_10_(*V*_*agg*_) collectively explained 81% of the variance in log_10_($\bar{{M}{a}{j}{A}{L}}$) (*r*^2^ = 0.81, *n* = 567*, p* <  < 0.01), with log_10_($\bar{\varepsilon }$) contributing 32% and log_10_ (*V*_*agg*_) 68% to this correlation.

Our findings show 97% of $\bar{{M}{a}{j}{A}{L}}$ values were below 0.1 cm in size (Fig. 1a), which is the size *L*_*k*_ for $\bar{\varepsilon }$* = *10^−6^ W kg^−1^ and the upper limit of dissipation rates typically observed in the ocean interior^30,31^. The positive correlation observed at length scales smaller than *L*_*k*_ demonstrates that turbulence enhancement of aggregation occurs at higher rate than disaggregation when shear is laminar^22,45^ and aggregate sizes are smaller than *L*_*k*_. This results in the net formation of larger aggregates. When the flow scale is equal to the Kolmogorov scale, the Reynolds number is 1 and the flow is very viscous, hence the resulting flow at this length scale is laminar shear^40^. Previous bottom boundary observations have shown a decrease in aggregates size when *L*_*k*_ was smaller than 0.1 cm, equivalent *ε* > 10^−6^ W kg^−1^^22^. Therefore, we expect that for $\bar{\varepsilon }$ > 10^−6^ W kg^−1^ and $\bar{{M}{a}{j}{A}{L}} < {L}_{k}$the disaggregation rate exceeds the aggregation rate, as shear associated with the smallest turbulent eddies causes breakup and inhibits further size increases.

**Figure 2:**
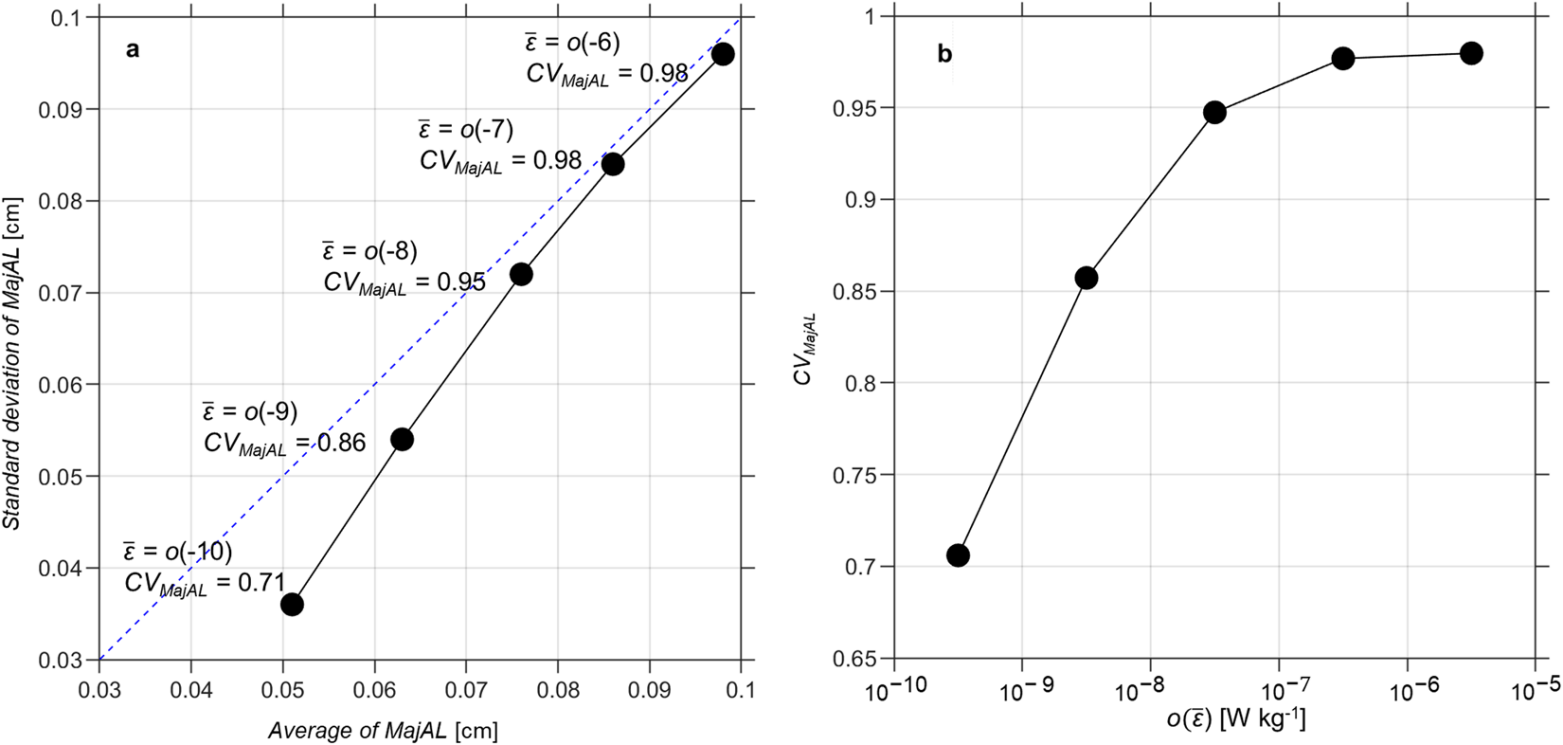
**(a)** Standard deviation versus mean size of aggregate for 5 orders of turbulent kinetic energy dissipation rate $({\boldsymbol{o}}(\bar{{\boldsymbol{\varepsilon }}}),\,W\,{\text{kg}}^{-1})$ and **(b)** *CV*_*MajAL*_ for each order of $o(\bar{{\boldsymbol{\varepsilon }}})$. (**a**) Aggregates were sorted into 5 turbulence ranges based on corresponding *ε*; individual aggregates within each range were used to calculate mean and standard deviation of *MajAL*. Blue dashed line indicates where *CV*_*MajAL*_, given by $\frac{\text{Standard}\,\text{deviation}}{{\rm{Mean}}}$, is 1. *CV*_*MajAL*_ and $o(\overline{\,\varepsilon })$ for each point are annotated. The total number of aggregates, average size and standard deviation and *CV*_*MajAL*_ for each turbulence range were: ($o(\overline{\,\varepsilon })$ = 10^−10^) 182493, 0.051, ± 0.036 cm, 0.71; ($o(\overline{\,\varepsilon })$ = 10^−9^) 164335, 0.063 ± 0.054 cm, 0.86; ($o(\overline{\,\varepsilon })$ = 10^−8^) 471271, 0.076 ± 0.072 cm, 0.95; ($o(\overline{\,\varepsilon })$ = 10^−7^) 284695, 0.086 ± 0.084 cm, 0.98 and ($o(\overline{\,\varepsilon })$ = 10^−6^) 166566, 0.098 ± 0.096 cm, 0.98, respectively. (**b**) *CV*_*MajAL*_ increased as $o(\overline{\,\varepsilon })$ increased.

While values of $\bar{{M}{a}{j}{A}{L}}$ shown in Fig. 1 were calculated using 10 m averages, the number of individual aggregates with *MajAL* larger than *L*_*k*_ remained relatively small. Above $\bar{\varepsilon }$ ≥ 10^−6^ W kg^−1^, 63% of individual *MajAL* sampled (non-averaged samples, *n* = 166,566) were smaller than *L*_*k*_. At lower turbulent intensities, the proportion of individual aggregates smaller than *L*_*k*_ increased from 80% at $\bar{\varepsilon }$ = *o*(10^−7^ W kg^−1^) to 99% of aggregates at $\bar{\varepsilon }$ = *o*(10^−10^ W kg^−1^). This shift demonstrates that aggregate size distribution is a dynamic property, with the potential for some aggregates to increase in size even under high average turbulent intensities ($\bar{\varepsilon }$ > 10^−6^ W kg^−1^) and for others to undergo disaggregation at lower average turbulent intensities ($\bar{\varepsilon }$ < 10^−6^ W kg^−1^). This interpretation is supported by results shown in Fig. 2, which demonstrates increases in the variability of individual aggregate sizes around the mean, expressed as the coefficient of variation, (*CV*_*MajAL*_ = standard deviation/mean), under increasing turbulent intensities.

For a more direct comparison between turbulence and aggregate size we calculated the mean size of individual aggregates for each order of magnitude of $\overline{\,\varepsilon }$($\bar{{{M}{a}{j}{A}{L}}_{\varepsilon }}$, cm). Increases in $\bar{{{M}{a}{j}{A}{L}}_{\varepsilon }}$ dropped from ~20% between $\bar{\varepsilon }$ = *o*(10^−10^ W kg^−1^) and *o*(10^−9^ W^−1^) to ~10% between $\bar{\varepsilon }$ = *o*(10^−7^ W kg^−1^) and *o*(10^−6^ W kg^−1^) (Fig. 2a) and were associated with a corresponding increase in the coefficient of variation (*CV*_*MajAL*_) from 0.69 to 0.98 (Fig. 2b). The plateau in *CV*_*MajAL*_ observed at higher turbulent intensities is consistent with disaggregation rates increasing as both turbulence levels and the average size of aggregates increase (Fig. 1a).

**Figure 3:**
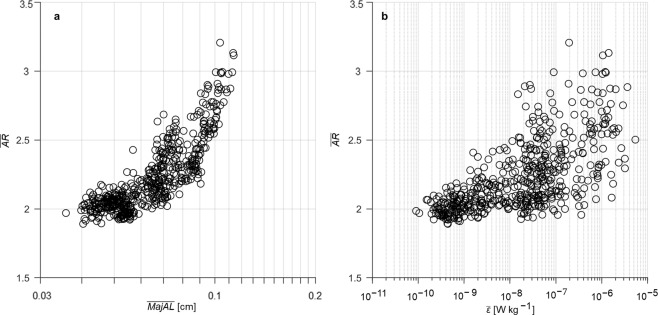
Changes in aggregate morphology with (**a**) aggregate size and (**b**) turbulence intensity. Relationship between the average aspect ratio ($\bar{{A}{R}}$) of aggregates (*n* = 567) with corresponding average values of; (**a**) major axis length ($\bar{{M}{a}{j}{A}{L}}$, cm) and (**b**) the turbulent kinetic energy dissipation rate ($\bar{\varepsilon },$ W kg^−1^). All values were averaged over 10 m depth intervals.

Increases in aggregate size with turbulence were also associated with changes in aggregate morphology (Fig. 3). The increase in ($\bar{{A}{R}})$ with log_10_($\bar{{M}{a}{j}{A}{L}}$) (Fig. 3a; *r*^2^ = 0.45, *n* = 567, *p* <  < 0.001) and log_10_ $\bar{\varepsilon }$ (Fig. 3b; *r*^2^ = 0.40, *n* = 567, *p < * < 0.001) shows that aggregates became elongated with increases in size and turbulence intensity. Numerical simulations and laboratory experiments^46–48^ have shown that inertial particles in turbulence cluster in regions of high-strain. Our results suggest that larger aggregates become inertial, possibly being strained by shear due to strong turbulence, resulting in elongation. Whilst increased inertial force on larger aggregates may also enhance breakage under strong turbulence, laboratory experiments^49^ and numerical simulations^50^ have demonstrated the settling velocity of elongated phytoplankton increase under elevated turbulence. It is possible that aggregate settling velocity increases due to both morphological changes (Fig. 3b) and size increases (Figs 1 and 2) under increasing turbulence up to a critical turbulent intensity, $\bar{\varepsilon }$* = *10^−6^ W kg^−1^.

**Figure 4:**
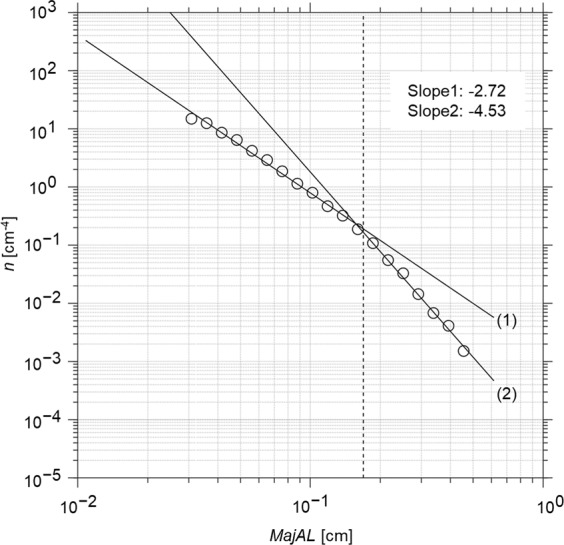
Aggregate number spectrum. Number spectrum (*n*, cm^−4^) shows the aggregate size distribution over logarithmically increasing *MajAL* (cm) size classes. Data were from the Kuroshio extension (37˚04′05″N, 142˚54′36″E) where maximum water depth exceeded 5000 m. All individual aggregates sampled in depths 10–100 m (total 17,526 aggregates) were used to construct *n*. Dashed line indicates *L*_*k*_ = 0.17 cm based on the cruise average dissipation rate ($\bar{{\varepsilon }_{{C}{A}}}$ = *o*(10^−7^ W kg^−1^). The fitted slopes are −2.72 (slope 1) and −4.53 (slope 2) and the intersection between the two lines *L*_*int*_ is 0.16 cm.

Aggregate number spectrum (*n*, cm^−4^)^43^ as a function of log_10_(*MajAL*) describes the non-averaged size distribution of individual aggregates throughout the water column. Figure 4 shows a spectrum for individual aggregates sampled between 10 and 100 m depth from the Kuroshio extension (37˚04′05″N, 142˚54′36″E). Here, an average dissipation rate *ε*_*CA*_ was computed from all individual *ε* to estimate the corresponding mean Kolmogorov scale (*L*_*k*,*CA*_, cm) in these near surface waters (Fig. 4, dashed lines). Two slopes were fitted to the number spectrum (Fig. 4, solid lines), with a gradient of −2.72 for the smaller aggregate size range (slope 1) and −4.53 for the larger aggregate size range (slope 2). There was a decrease in the number of aggregates expected by using the line fit to the smaller aggregates for aggregates larger than the intersection (*L*_*int*_, cm) of the two lines. Here, *L*_*int*_ is 0.16 cm and the Kolmogorov scale based on *ε*_*CA*_ is *L*_*k*,*CA*_ = 0.17 cm. The ratio between *L*_*int*_ and *L*_*k*,*CA*_is 0.95 and shows the number of aggregates decreases significantly when aggregate size is larger than *L*_*k*,*CA*_. This trend was consistent across all campaigns used in this study. The significant decrease in *n* as the aggregate size exceeds *L*_*k*,*CA*_ suggests that the role of particle collision in aggregate formation becomes smaller as disaggregation due to turbulence becomes more prominent.

**Figure 5:**
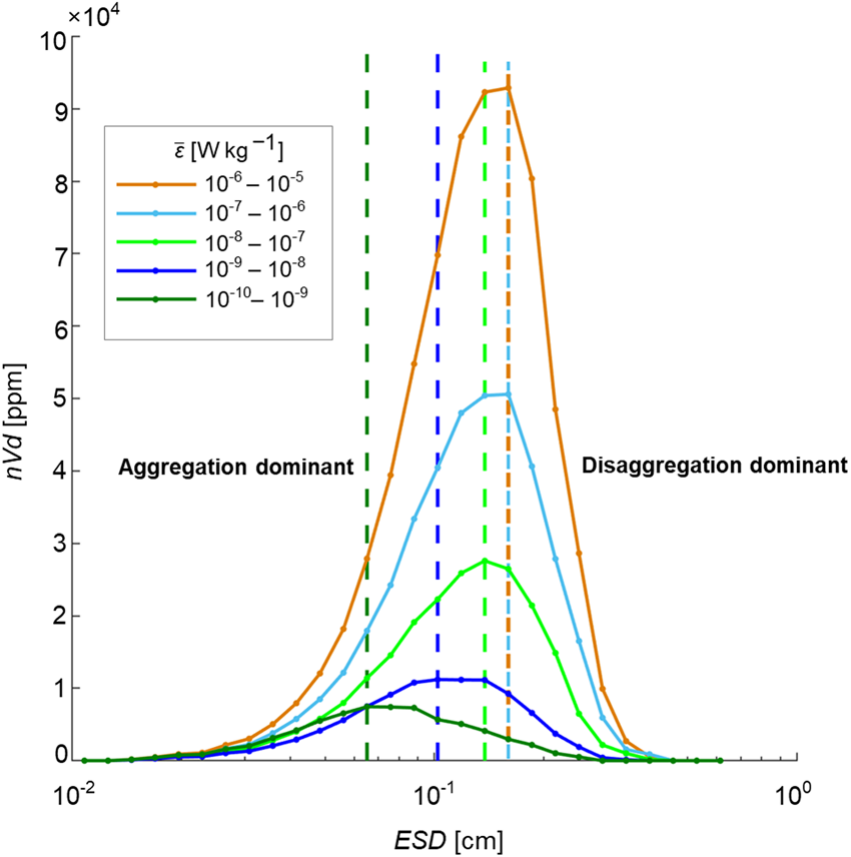
Turbulent mediation of aggregation and disaggregation rates in the upper ocean. Changes in the normalised volume distribution (*nVd*, ppm) of aggregates as a function of equivalent spherical diameter (*ESD*, cm) for 5 orders of turbulent kinetic energy dissipation rate $(o(\bar{\varepsilon }),W\,\text{kg}-1)$ measured in the upper (10–100 m) ocean. Vertical dashed lines indicate the mode for each lognormal *nVd* distribution. Aggregation dominated below the mode and disaggregation above the mode.

The normalized volume distribution (*nVd*, ppm)^43,44^ as a function of log_10_(*ESD*) provides further insight into aggregation and disaggregation processes (Fig. 5). The shape of the *nVd* distributions is similar to the lognormal distributions described previously^43^. A simulation^51^ showed that *nVd* for aggregates have a lognormal-like distribution when both aggregation and disaggregation are taken into account, suggesting that disaggregation occurs at all level of turbulence (Fig. 5). This is consistent with the original breakage model proposed by Kolmogorov^52^. Lognormal turbulence theory^52,53^ shows that a fraction of the water over which $\bar{\varepsilon }$ is calculated contains localized regions of the turbulent kinetic energy dissipation rate higher than ensemble average^54^. Hence, for the range of observed $\bar{\varepsilon }$, parcels of highly localized turbulence may exceed *ε* = 10^−6^ W kg^−1^ and are expected to cause disaggregation even under conditions of low average dissipation rates. The area under the curve of *nVd* is proportional to *V*_*agg*_^43,44^. Increased *nVd* with $\bar{\varepsilon }$ is consistent with the aggregation rate increasing under stronger turbulence. The distribution peak shifted to larger *ESD* with increasing in $\bar{\varepsilon }$ by ~15–20% when $\bar{\varepsilon }$ increased one order of magnitude. The increase was limited to ~0.16 cm when $\bar{\varepsilon }$ = 10^−6^ – 10^−5^ W kg^−1^. For *ESD* larger than the distribution peak, negative *nVd* slopes indicate that loss of the large aggregates by disaggregation counters their production by aggregation; steeper slopes at higher $\bar{\varepsilon }$ show that the loss becomes more rapid as the turbulence intensity increases. This is consistent with turbulence-induced disaggregation rate overtaking the aggregation rate with increased *V*_*agg*_.

## Conclusions

Our observations provide a comprehensive set of simultaneous measurements of aggregate concentrations as a function of size that resolve the full range of turbulent intensities, $\bar{\varepsilon }$ = 10^−10^ – 10^−6^ W kg^−1^, found within the upper ocean away from energetic near surface and bottom boundary layers (~10–100 m depth). Although turbulent intensities in coastal environments and near boundary layers can far exceed 10^−5^ W kg^−1^, our observed values cover the range of intensities found typically over the majority of worlds upper ocean surface layer^30,31^. Our direct observations show turbulence enhances aggregation up to ~$\bar{\varepsilon }$ = 10^−6^ W kg^−1^ with greater turbulence intensities cause increasing disaggregation, consistent with laboratory^13^ and theoretical^14,17^ studies and the early conceptual view of aggregation dynamics studied in coastal environments^15^. Since most of the ocean upper water column interior contains $\bar{\varepsilon }$ < 10^−6^ W kg^−1^^30,31^, turbulent mediation of aggregate size and morphology is likely to be an important factor influencing a range of biogeochemical processes, including carbon sequestration. This is because aggregate size and morphology are important determinant factors of settling velocities and carbon flux^7,55^,microbial abundances^56,57^ and associated biogeochemical activity through bacterial remineralization^1,4,56^. As climate change is expected to supress turbulence intensity^8^ and alter phytoplankton communities^32,33^ in the euphotic zone, the mediation of aggregates by turbulence may have unexpected consequences for global carbon cycle via the biological pump^9,58^.

## Data Availability

All data used in this study are available from the first author and corresponding author (M.T. and H.Y.) upon request to jasmine222mari@gmail.com or hide@kaiyodai.ac.jp.
